# Supplementary material for: Intron-containing RNA from the HIV-1 provirus activates type I interferon and inflammatory cytokines
Source: Nat Commun. 2018 Dec 13;9:5305. doi: 10.1038/s41467-018-07753-2 (PMC6294009; doi:10.1038/s41467-018-07753-2)
Supplement: Supplementary file 1 — Supplementary Information [file 41467_2018_7753_MOESM1_ESM.pdf]

Supplement for:

Intron-containing RNA from the HIV-1 provirus activates type I interferon and inflammatory cytokines

Sean Matthew McCauley, Kyusik Kim, Anetta Nowosielska, Ann Dauphin, Leonid Yurkovetskiy, William Edward Diehl, and Jeremy Luban

Including:

Supplementary Figures 1, 2, 3, and 4 with corresponding Figure Legends.

Supplementary Tables 1, 2, 3, 4

Supplementary Figure 1

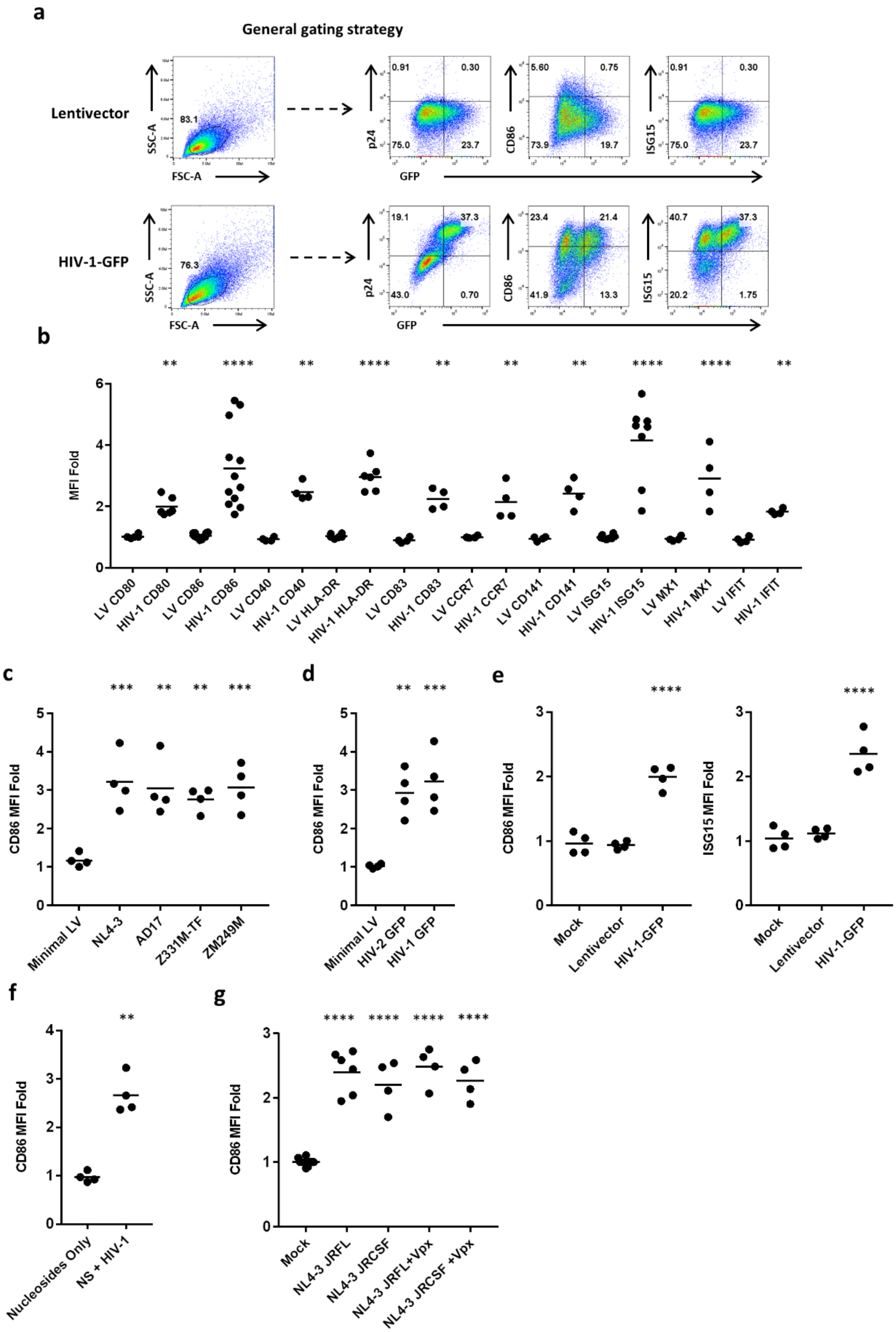

Supplementary Figure 1

a, Gating strategy for all flow cytometry data presented in this manuscript. **b**, Data for Figure 1b and 1c showing the mean fluorescence intensity of various markers of innate activation in DCs transduced with HIV-1-GFP vs minimal lentivector. **c,** Data for Figure 1d of CD86 MFI on DCs transduced with several single cycle molecular clones. **d,** Data for Figure 1e of DC transductions of HIV-2-GFP single cycle vector. **e.** Data for Figure 1f of DCs treated with the supernatant of autologous DCs transduced with HIV-1 vectors. **f,** Data for Figure 1g of DC HIV-1-GFP transductions using nucleosides to enhance infectivity. **g,** Data for Figure 1h of spreading infections of Mac tropic or T cell tropic HIV-1 on DCs treated with or without Vpx. Significance was calculated in all cases by one-way ANOVA with Dunnett's post-test comparing test against minimal lentivector negative. (* = $p \leq 0.05$, ** = $p \leq 0.01$, *** = $p \leq 0.001$, **** = $p \leq 0.0001$).

Supplementary Figure 2

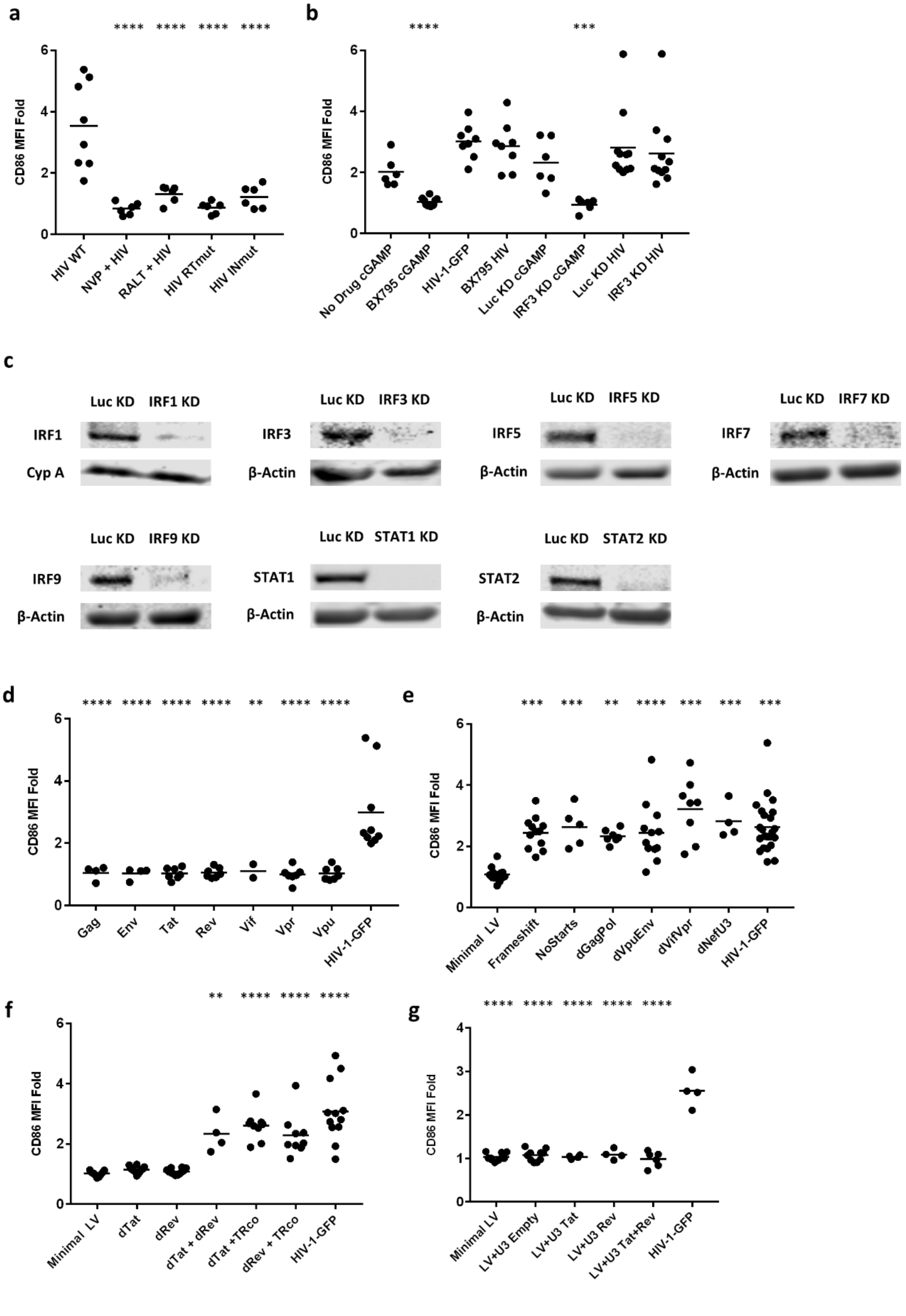

Supplementary Figure 2

a, Data for Figure 2a showing the mean fluorescence intensity of CD86 of DC transduced with HIV-1 RT or IN mutants or inhibitors to RT and IN. **b,** Data for Figure 2b of DC TBK1 inhibition or DC IRF3 knockdowns challenged with either cGAMP or HIV-1-GFP. **c,** Western blots of DC knockdowns for IRF1, 3, 5, 7, 9 and STAT 1 and 2. **d,** Data for Figure 2d of DC transduced with minimal lentivectors expressing HIV-1 genes. **e,** Data for Figures 2e and 2f of DC transductions of HIV-1 mutants and truncations. **f,** Data for Figure 2g of DCs transduced with HIV-1 containing mutations to Tat or Rev as well as their rescue *in trans*. **g,** Data for Figure 2h of DCs transduced with minimal lentivectors designed to transcribe from the HIV-1 LTR. Significance was calculated in all cases by one-way ANOVA with Dunnett's post-test comparing test against minimal lentivector negative. (* = $p \leq 0.05$, ** = $p \leq 0.01$, *** = $p \leq 0.001$, **** = $p \leq 0.0001$).

Supplementary Figure 3

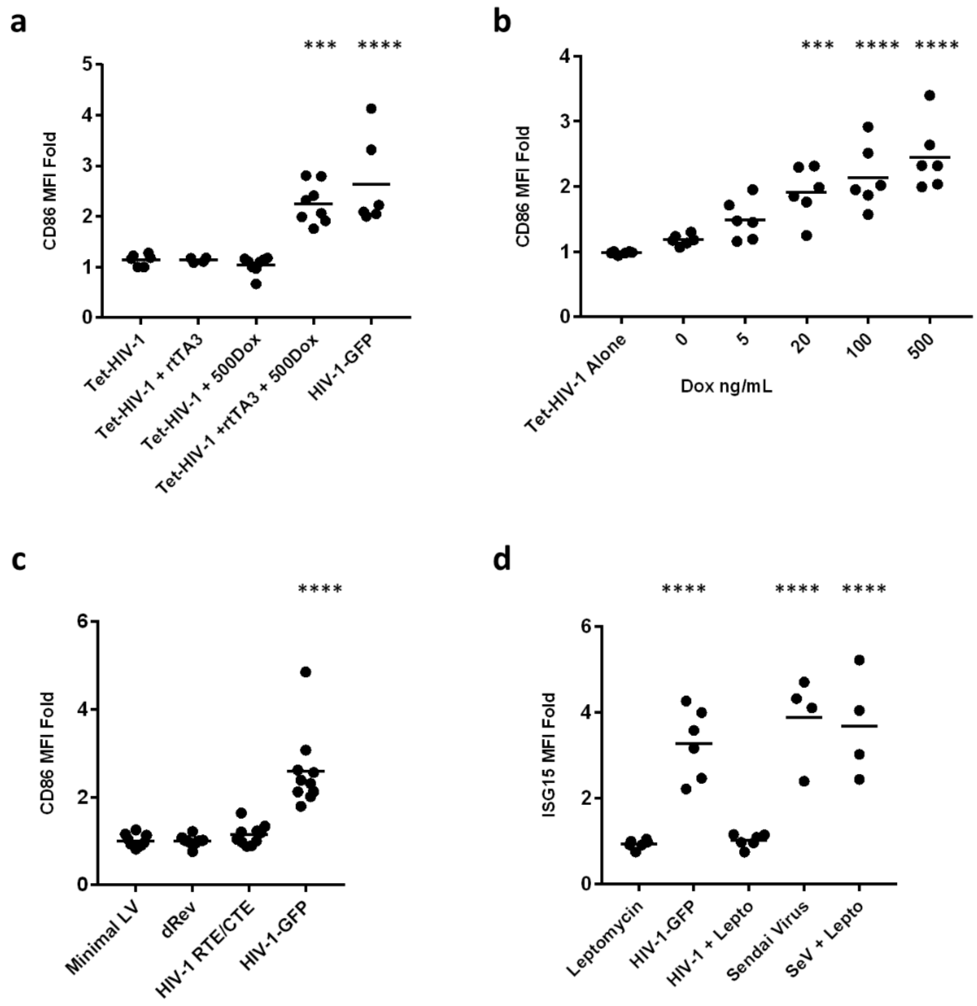

Supplementary Figure 3

a, Data for Figure 3a of CD86 MFI on DCs transduced with Tet-HIV-1 with and without rtTA3 *in trans* and doxycycline. **b,** Data for Figure 3b of doxycycline titration on Tet-HIV-1 and rtTA3 transduced DCs. **c,** Data for Figure 3c of DCs transduced with HIV-1 containing the RTEm26CTE Tap1 export element. **d,** Data for Figure 3d and 3e of ISG15 MFI on DCs treated with or without Leptomycin and challenged with HIV-1-GFP or Sendai virus. Significance was calculated in all cases by one-way ANOVA with Dunnett's post-test comparing test against minimal lentivector negative. (* = $p \leq 0.05$, ** = $p \leq 0.01$, *** = $p \leq 0.001$, **** = $p \leq 0.0001$).

Supplementary Figure 4

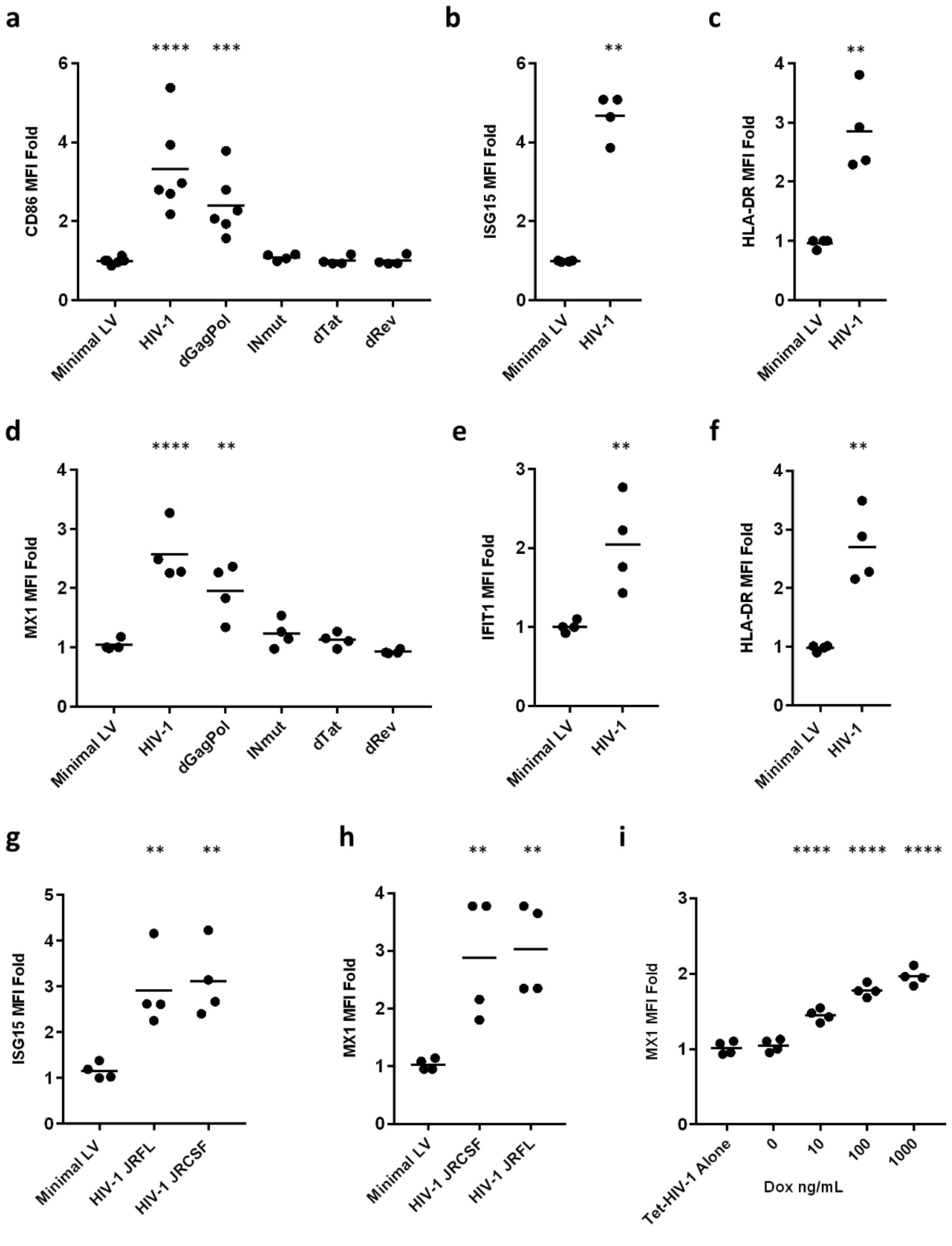

Supplementary Figure 4

a, Data for Figure 4a and 4b of CD86 MFI of Macrophages transduced with HIV-1-GFP and select mutants. **b,** Data for Figure 4a of ISG15 MFI on HIV-1-GFP transduced Macrophages. **c,** Data for Figure 4a of HLA-DR MFI on HIV-1 transduced Macrophages **d,** Data for Figure 4a and 4b of MX-1 MFI of CD4+ T cells transduced with HIV-1-GFP and select mutants. **e,** Data for Figure 4a of MX-1 IFIT1 on HIV-1-GFP transduced CD4+ T cells. **f,** Data for Figure 4a of HLA-DR MFI on HIV-1-GFP transduced CD4+ T cells. **g,** Data for Figure 4c of ISG15 MFI of spreading infections of Mac tropic or T cell tropic HIV-1 on CD4+ T cells. **h,** The same samples as in "**g**" but of MX1 MFI. **i,** Data for Figure 4d of MX1 MFI of doxycycline titration on CD4+ T cells transduced with Tet-HIV-1 and rtTA3. Significance was calculated in all cases by one-way ANOVA with Dunnett's post-test comparing test against minimal lentivector negative. (* = $p \leq 0.05$, ** = $p \leq 0.01$, *** = $p \leq 0.001$, **** = $p \leq 0.0001$).

Supplementary Table 1. Plasmids used in this study.

Plasmid Name	Purpose	Notes	Addgene code #
pBS NL4-3 *env*^{FS} eGFP	Single-cycle, full-length HIV-1	"HIV-1-GFP" in the manuscript. HIV-1 NL4-3 in pBluescript, flanking host sequences deleted, frameshift in *env*, eGFP in place of *nef*[11]	101317
pUC57mini NL4-3 Δ*env* eGFP	HIV-1 clade B molecular clone	Molecular clone of NL4-3 with deletion of 79 nucleotides following the Env signal peptide and eGFP in place of *nef*	101318
pUC57mini AD17 Δ*env* eGFP	HIV-1 clade B molecular clone	Molecular clone of transmitted/founder virus HIV-1_{AD17}[20] with deletion of 79 nucleotides following the Env signal peptide and eGFP in place of *nef*	101319
pUC57mini Z331M-TF Δ*env* eGFP	HIV-1 clade C molecular clone	Molecular clone of transmitted/founder virus HIV-$1_{Z331M\text{-}TF}$[21] with deletion of 79 nucleotides following the Env signal peptide and eGFP in place of *nef*	101320
pUC57mini ZM249M Δ*env* eGFP	HIV-1 clade C molecular clone	Molecular clone of transmitted/founder virus HIV-1_{ZM249M}[22] with deletion of 79 nucleotides following the Env signal peptide and eGFP in place of *nef*	101321
pMD2.G	VSV G	Pseudotype HIV-1 vectors with VSV G	12259
psPAX2	HIV-1 *gag-pol*	"3-part" lentivector or for complementation of assembly-incompetent HIV-1 vectors	12260
SIVgpx	SIV_{MAC251} *gag-pol*/*vpx*	Production of SIV VLPs containing Vpx protein	101322
pALPS[12]	Minimal lentivector	Includes *cis*-acting elements required for reverse transcription and integration, psi RNA packaging element, RRE, cPPT, PPT, mutation in the 3'LTR U3 that eliminates LTR-based transcription, and SFFV promoter to express genes of interest.	90996
pALPS eGFP	eGFP lentivector	Encodes eGFP	101323
pALPS *gag*	*gag* lentivector	Encodes codon optimized NL4-3 *gag*	101324
pALPS *env*	*env* lentivector	Encodes codon optimized JR-CSF *env*	101325
pALPS *tat*	*tat* lentivector	Encodes codon optimized NL4-3 *tat*	101326
pALPS *rev*	*rev* lentivector	Encodes codon optimized NL4-3 *rev*	101327
pALPS *vif*	*vif* lentivector	Encodes codon optimized NL4-3 *vif*	101328
pALPS *vpr*	*vpr lentivector*	Encodes codon optimized NL4-3 *vpr*	101329
pALPS *vpu*	*vpu lentivector*	Encodes codon optimized NL4-3 *vpu*	101330
pALPS *tat*-P2A-*rev*	*tat* and *rev lentivector*	Lentivector expressing codon optimized NL4-3 *tat* and *rev* linked by P2A peptide coding sequence (GSGATNFSLLKQAGDVEENPGP)	101331
pBS NL4-3 *env*^{FS} eGFP RT-D185K/D186L[25]	RT mutant	pBS NL4-3 *env*^{FS} eGFP with mutation that disrupts RT catalytic activity	101332

pBS NL4-3 *env*^{FS} eGFP IN-D116A[25]	IN mutant	pBS NL4-3 *env*^{FS} eGFP with mutation that disrupts IN catalytic activity	101333
pBS NL4-3 *env*^{FS} eGFP PR-D25A[25]	PR mutant	pBS NL4-3 *env*^{FS} eGFP with mutation that disrupts Protease catalytic activity	101334
pALPS puro miR30-L1221[12]	Luciferase knockdown	negative control for knockdowns target site: CTTGTCGATGAGAGCGTTTGT	101335
pALPS puro miR30-IRF1[12]	IRF1 knockdown	Target site TTGCTCTTAGCATCTCGGCTG	117156
pALPS puro miR30-IRF3[12]	IRF3 knockdown	Target site ATCAGATCTACAATGAAGGGC	101336
pALPS puro miR30-IRF5[12]	IRF5 knockdown	Target site TATTTCCCTGTCTCCTTGGCC	117157
pALPS puro miR30-IRF7[12]	IRF7 knockdown	Target site ATAAGGAAGCACTCGATGTCG	117158
pALPS puro miR30-IRF9[12]	IRF9 knockdown	Target site AATTATCACAAAGAGGACAGG	117159
pALPS puro miR30-STAT1[12]	STAT1 knockdown	Target site ATATCCAGTTCCTTTAGGGCC	117160
pALPS puro miR30-STAT2[12]	STAT2 knockdown	Target site TTTAAGTTCCACAGACTTGGA	117161
pALPS puro miR30-TAK1[12]	TAK1 knockdown	Target site AGCGCCCTTCAATGGAGGAAAT	117162
pALPS_3'LTR GFP@gag start SFFV-	U3+ lentivector GFP at *gag* start SFFV promoter	Repaired U3 allows LTR-based transcription by the provirus with GFP as a marker for expression. WPRE was deleted.	101337
pALPS_3'LTR GFP@*gag* start SFFV *tat*	U3+ lentivector GFP at *gag* start SFFV-*tat*	LTR drives GFP and internal SFFV promoter drives codon optimized *tat*	101338
pALPS_3'LTR GFP@*gag* start SFFV *rev*	U3+ lentivector GFP at *gag* start SFFV-*rev*	LTR drives GFP and internal SFFV promoter drives codon optimized *rev*	101339
pALPS_3'LTR GFP@*gag* start SFFV *tat*-P2A-*rev*	U3+ lentivector with SFFV-*tatP2Arev*	LTR drives GFP and internal SFFV promoter drives codon optimized *tat* and *rev*.	101340
pNL4-3 *env*^{FS} eGFP *gag*^{2xFS}	No Gag synthesis	1st frameshift is CG nucleotide insertion in MA at nt 832. 2nd is a CTAG addition in CA at nt 1508.	101341
pNL4-3 *env*^{FS} eGFP NoStarts	No Gag synthesis	All ATGs from the start of *gag* to NC mutated to ATC except the first which was mutated to ACG	101342
pBS NL4-3 *env*^{FS} eGFP Δ*gag/pol*	*gag-pol* deletion	Deletion from the start of *gag* until 229 bases before the cPPT	101343

pNL4-3 *env*^{FS} eGFP Δ*vif/vpr*	*vif*/*vpr* deletion	Deletion from NL4-3 nt 5582-6199 encompassing Vif and Vpr coding sequence	101344
pNL4-3 *env*^{FS} eGFP Δ*vpu/env*	*vpu*/*env* deletion	Deletion from NL4-3 nt 6054-7489 encompassing all of *vpu* and *env* until before the RRE	101345
pBS NL4-3 *env*^{FS} eGFP Δ*nef*/U3	*nef*/U3 deletion	Deletion from NL4-3 nt 8911-9022 and 9088-9377. This deletes *nef* and U3 LTR sequences	101346
pBS NL4-3 *env*^{FS} eGFP 5'CMV Δ*tat*	HIV-1 with inactivating mutations in *tat*	*tat* ATG->ACG (silent in *vpr* reading frame), nt 78 mutated T->G to change Tyr to stop codon, nt 116 mutated T->C to disrupt Met, 5'LTR replaced with CMV-R-U5 from pALPS for *tat*-independent transcription in HEK293E cells.	101347
pBS NL4-3 *env*^{FS} eGFP Δ*rev*	HIV-1 with inactivating mutations in *rev*	All mutations in *rev* are silent with respect to the *tat* reading frame. Start ATG->ACG and nts 68-71 were mutated AGC->TCA to change tyrosine to a stop.	101348
pBS NL4-3 *env*^{FS} eGFP 5'CMV Δ*tat* ΔTARx2_d2TetOp[28]	Tet-inducible, *tat*-independent HIV-1	2xTet Operator inserted between NFkB and Sp1 sites in U3 of HIV-1 Δ*tat* with 5' CMV-R-U5. 5' and 3' TAR elements were mutated to: 5'-GGTCTCTCTGGTTAGACCAGAAAGGAGCATTGGAGCTCTCTGGCTAACTAGGGAACCC-3'	101349
pALPS rtTA3_V14[28]	rtTA3 lentivector	Codon optimized rtTA3 used *in trans* with Tet inducible HIV-1	101350
pSC101 NL4-3 *env*^{FS} Δ*rev* ΔRRE RTEm26CTE[29]	Rev (CRM1) independent HIV-1	HIV-1 ΔRev was cloned into pSC101 and modified to include an RTEm26CTE element in order to utilize the NXF1 RNA export pathway. The RRE was also mutated.	101351

Supplementary Table 2. Drugs and reagents.

Drug	Action	Source	Working concentration	HIV-1 DC maturation
Doxycycline	rtTA3 activator	Sigma (D9891)	10-1000 ng/mL	-
cGAMP	STING activator	Invivogen (tlrl-nacga23)	25 µg/mL	-
PHA-P	T cell mitogen	Sigma (L1668)	5 µg/mL	-
2′deoxyguanosine monohydrate	For nucleoside assisted transductions	Sigma (D0901)	2 mM	-
2’ deoxythymidine	For nucleoside assisted transductions	Sigma (T1895)	2 mM	-
2′deoxyadenosine monohydrate	For nucleoside assisted transductions	Sigma (D8668)	2 mM	-
2′deoxycytidine hydrochloride	For nucleoside assisted transductions	Sigma (D0776)	2 mM	-
Sendai Virus (SeV) Cantell strain	Challenge virus	Charles River Labs (VR-907)	200 HA units/mL	-
Nevirapine	Reverse transcriptase inhibitor	NIH AIDS reagent program (4666)	5 µM	Inhibits
Raltegravir	Integrase inhibitor	NIH AIDS reagent program (11680)	10 µM	Inhibits
Leptomycin	CRM1 inhibitor	Invivogen (tlrl-lep)	25 nM	Inhibits
Cyclosporin A	Cyclophilin A inhibitor	Sigma (30024)	5 µM	No effect
GW-5075	c-Raf	Sigma (G6416)	1, 5, 25 µM	No effect
BAY11-7082	IkB-a Inhibitor	Invivogen (tlrl-b82)	1, 2.5, 10 µM	No effect
U0126	MEK1 and MEK2 Inhibitor	Invivogen (tlrl-u0126)	10, 25, 50 µM	No effect
SB203580	p38 MAP Kinase Inhibitor	Invivogen (tlrl-sb20)	1, 2.5, 10 µM	No effect
MCC950	NLRP3-inflammasome inhibitor	Invivogen (inh-mcc)	1, 2.5, 10 µM	No effect
SP600125	JNK Inhibitor	Invivogen (tlrl-sp60)	10, 25, 100 µM	No effect

Z-VAD-FMK	Pan-Caspase Inhibitor	Invivogen (tlrl-vad)	1, 5, 20 μM	No effect
NQDI-1	ASK1 inhibitor	Sigma (SML0185)	1, 10, 100 μM	No effect
ISRIB	eIF2a phosphorylation inhibitor	Sigma (SML0843)	1, 10, 100 μM	No effect
VX-765	Caspase 1 inhibitor	Invivogen (inh-vx765i)	1, 10, 100 μM	No effect
Dexamethasone	NF-kB and MAPK inhibitor	Invivogen (tlrl-dex)	10, 100, 1000 nM	No effect
Chloroquine	inhibitor of endosomal acidification	Invivogen (tlrl-chq)	1, 10, 100 μM	No effect
Amlexanox	TBK1/IKKε inhibitor	Invivogen (inh-amx)	1, 10, 100 ug/mL	No effect
BX795	TBK1/IKKε inhibitor	Invivogen (tlrl-bx7)	0.5, 1, 2 μM	No effect
NG25 trihydrochloride	TAK1 & LYN, MAP4K2 and Abl inhibitor	Sigma (SML1332)	50, 100, 500, 1000 nM	No effect
5Z-7-Oxozeaenol	TAK1 & MAP4K2 inhibitor	Sigma (O9890)	50, 100, 500, 1000 nM	No effect
C16	PKR inhibitor	Sigma (I9785)	1 μM	No effect
2AP	PKR inhibitor	Invivogen (tlrl-apr)	5 μM	No effect

Supplementary Table 3. qRT-PCR probes.

RNA Target	Taqman probe ID#
CXCL10	Hs00171042_m1
IFNB1	Hs01077958_s1
IL15	Hs01003716_m1
OAZ1	Hs00427923_m1
ACTB	Hs01060665_g1

Supplementary Table 4. Antibodies used in this study.

Target antigen	Clone	Fluorophore	Source	Dilution/ Concentraiton
CD80	2D10	PE, APC	Biolegend	1:100
CD86	IT2.2	PE, APC	Biolegend	1:100
CD40	HB14	APC	Biolegend	1:100
HLA-DR	L249	PerCP, APC	Biolegend	1:100
CD83	HB15e	FITC, APC	Biolegend	1:100
CCR7	G043H7	PE	Biolegend	1:100
CD141	M80	PE	Biolegend	1:100
ISG15	#851701	PE, APC	R&D Systems	1:100
MX1	EPR19967	N/A	Abcam	1:100
IFIT	OTI3G8	N/A	Abcam	1:100
p24	KC57	FITC, PE	BeckmanCoulter	1:200
CD1a	HI149	FITC, PerCP	Biolegend	1:100
CD1c	L161	PE	Biolegend	1:100
CD14	HCD14	FITC, Pe-Cy7	Biolegend	1:100
CD11b	ICRF44	PE	Biolegend	1:100
CD11c	3.9	PE, PE-Cy7, APC	Biolegend	1:100
CD209 (DC-SIGN)	9E9A8	APC	Biolegend	1:100
CXCL10	JO34D6	PE	Biolegend	1:100
HLA-ABC	W6/32	FITC	Biolegend	1:100
CD3	OKT3	FITC, APC, BV650	Biolegend	1:100
CD4	OKT4	FITC, APC, Alexa700	Biolegend	1:100
Mouse-IgG	Poly4053	PE, APC	Biolegend	1:100
Rabbit-IgG	Poly4064	PE, AlexaFluor647	Biolegend	1:100
IRF1	13H3A44	N/A	Biolegend	1 ug/mL

IRF3	11904S	N/A	Cell Signal Tech	1:1000
IRF5	5A3A39	N/A	Biolegend	1 ug/mL
IRF7	12G9A36	N/A	Biolegend	1 ug/mL
IRF9	5A3A39	N/A	Biolegend	1 ug/mL
STAT1	9176S	N/A	Cell Signal Tech	1:1000
STAT2	4594S	N/A	Cell Signal Tech	1:1000
